# Supplementary material for: Structure–dynamics decoupling in soft-colloid suspensions
Source: Nat Commun. 2025 Dec 17;17:27. doi: 10.1038/s41467-025-66565-3 (PMC12764882; doi:10.1038/s41467-025-66565-3)
Supplement: Supplementary file 1 — Supplementary Information [file 41467_2025_66565_MOESM1_ESM.pdf]

Supplementary Information for:
Structure-Dynamics decoupling in soft-colloid suspensions

Adrián Arenas-Gullo[1,2], Joaquím Clara-Rahola[3], Phil N. Segré[4], José Ruiz-Franco[1,2], and Alberto Fernandez-Nieves[1,2,5]

[1] *Department of Condensed Matter Physics, University of Barcelona, Carrer de Martí i Franqués 1, Barcelona, 08028, Spain*

[2] *Institute of Complex Systems (UBICS), University of Barcelona, Carrer de Martí i Franqués 1, Barcelona, 08028, Spain*

[3] *HN Captial Consulting SL, Carrer del Mestre Francesc Civil 8, Girona, 17005, Spain*

[4] *Emory Oxford College, Emory St 810, Oxford, GA 30054, United States and*

[5] *ICREA-Institució Catalana de Recerca i Estudis Avançats, Passeig de Lluís Companys 23, Barcelona, 08010, Spain*

CONTENTS

SI. Position of the structure factor peak and stretching exponent in experiments 2

SII. Correlation function analysis 3

SIII. Experimental results for $\zeta = 3.0$ 4

SIV. Mapping experiments to simulation model 4

SV. Static-dynamic decoupling in simulations for other values of U_{mid} 5

SVI. Fitting parameter values from the modified Arrhenius plot 6

SVII. The $\zeta - \phi$ connection and the approach to the glass 7

References 8

SI. POSITION OF THE STRUCTURE FACTOR PEAK AND STRETCHING EXPONENT IN EXPERIMENTS

The inverse of the position of the $S(q)$ peak, $1/q^*$, scales linearly with R_h^{eff}, as shown in Fig. S1(a) for $\zeta = 1.5$ and in Fig. S1(c) for $\zeta = 3.0$. Linear fits of the data reveal a slope close to π, demonstrating that the peak of the structure factor reflects the particle size at these relatively high particle concentrations, and that normalizing the scattered intensity with the form factor measured in dilute condition does not appreciably change the expected shift of q^*. This further highlights that better estimates of the particle form factor, considering that the particles would have slightly shrunk for $\zeta > 1$ and that their morphology may have additionally changed would not have any qualitatively relevant effect in our data and subsequent interpretations.

The stretching parameter p, obtained from the fits of the correlation functions, exhibit no q-dependence, as shown in Fig. S1(b) for $\zeta = 1.5$ and in Fig. S1(d) for $\zeta = 3.0$. This holds at all temperatures. Note that $p \approx 1$ indicates diffusive behavior, while $p < 1$ indicates that the dynamics is subdiffusive and well captured by a stretched exponential behavior. For T corresponding to the softest states, $p \approx 1$, while $p < 1$ for temperatures where the particles are less soft. This in turn seems to correlate with whether the structure and dynamics couple or not.

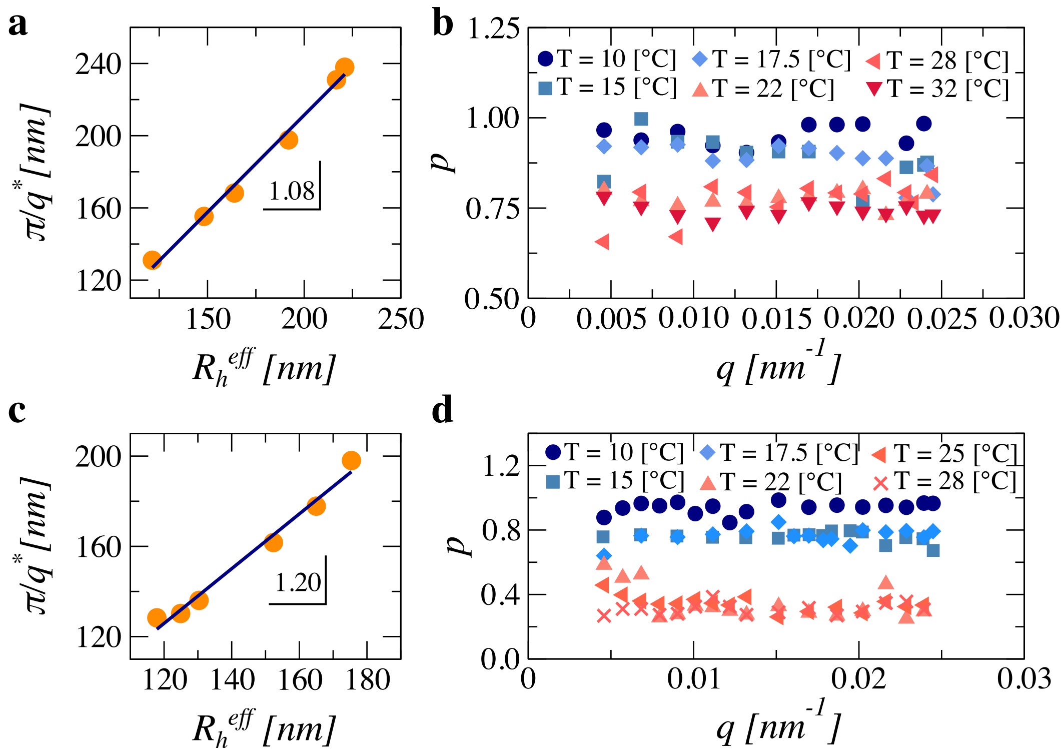

Figure S1: q^* **and** p **in experiments. a**,**c**, Linear dependence of $1/q^*$, with q^* the position of the $S(q)$ peak, with R_h^{eff}. Solid lines are linear fits to the data. The slope is in both cases close to π. **b**,**d** Stretching exponent p versus q for different T. In (**a**,**b**) $\zeta = 1.5$, while in (**c**,**d**) $\zeta = 3.0$.

SII. CORRELATION FUNCTION ANALYSIS

The correlation function for $\zeta = 1.5$ and $T = 22°C$, at $qR_h^{eff} = 3.31$, is shown in Fig. S2(a,b) as an example representative of the data obtained at other temperatures and other scattering wave vectors at this ζ. It exhibits a single decay to zero that is well described by a stretched exponential, shown with a line in the same figures. After two logarithmic transformations, the linear part allows accurately obtaining the stretching exponent p and the characteristic structural relaxation time τ_α.

For $\zeta = 3.0$ and various temperatures, the correlation function exhibits two decays. The case for $T = 25°C$ and $qR_h^{eff} = 3.01$ is shown in Fig. S2(c,d) as an example. In these cases, after a double logarithmic transformation, the data exhibits two linear regimes. We are particularly interested in the long-time decay, as it is associated to the structural relaxation of the suspension. The corresponding linear fit allows obtaining p and τ_α.

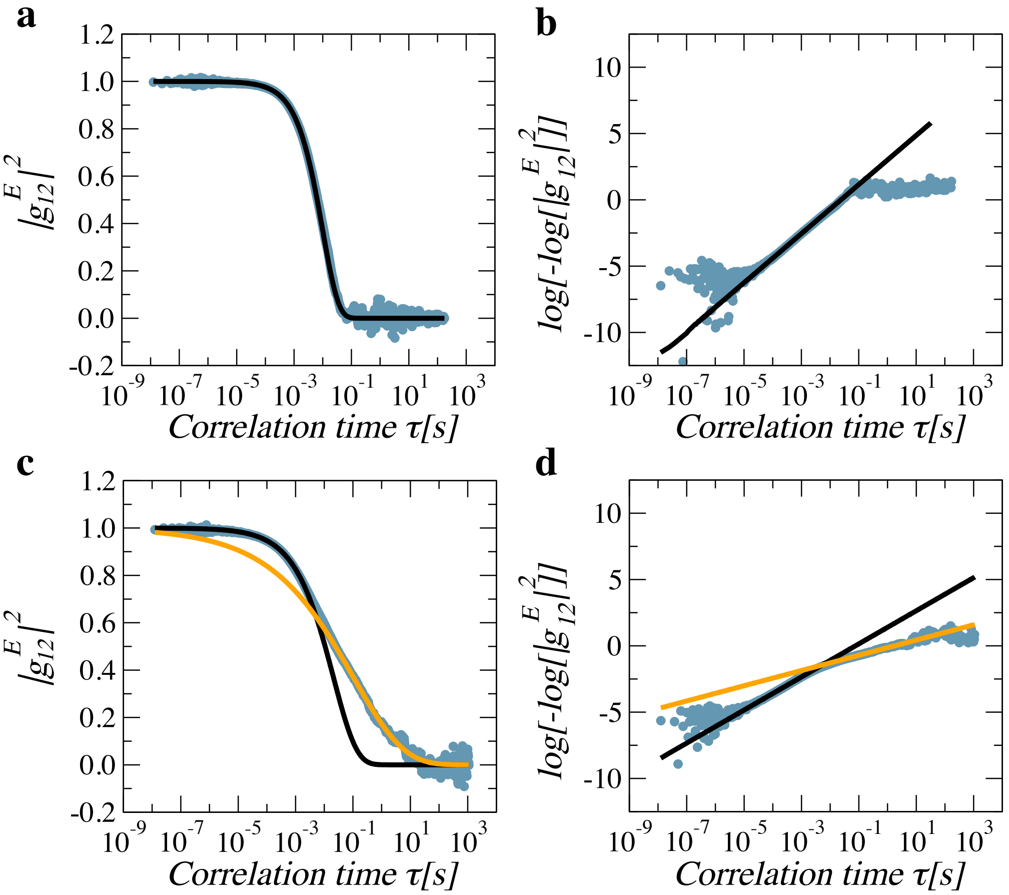

Figure S2: Example experimental correlation functions. a,b, $\zeta = 1.5$, $T = 22°C$ and $qR_h^{eff} = 3.31$. **c,d** $\zeta = 3.0$, $T = 25°C$ and $qR_h^{eff} = 3.01$. Symbols correspond to experimental data, while solid lines are fits of the data to (**a**) a single stretched exponential or (**c**) a sum of two stretched exponentials describing the short-time and long-time dynamics. Panels (**b**,**d**) are linearization plots of the data in panels (**a**,**c**) that allow obtaining both the stretching exponent and relaxation time.

SIII. EXPERIMENTAL RESULTS FOR $\zeta = 3.0$

We have also performed measurements at $\zeta = 3.0$. In this case, decoupling is only observed at $T = 10^o$C, as shown in Fig. S3(a). At higher temperatures, the dynamics qualitatively follows the structure, as also shown in Fig. S3(a). The correlation function is well described by a single stretched exponential at the lowest temperatures, while two stretched exponentials are required for higher T; this can be seen in Fig. S3(b). For stiffer particles, the dynamics becomes distinct at short and long times, reflecting caging and the associated structural relaxation. In this case, we are mostly interested in the decay at long times, which is the one that allows us to obtain τ_α.

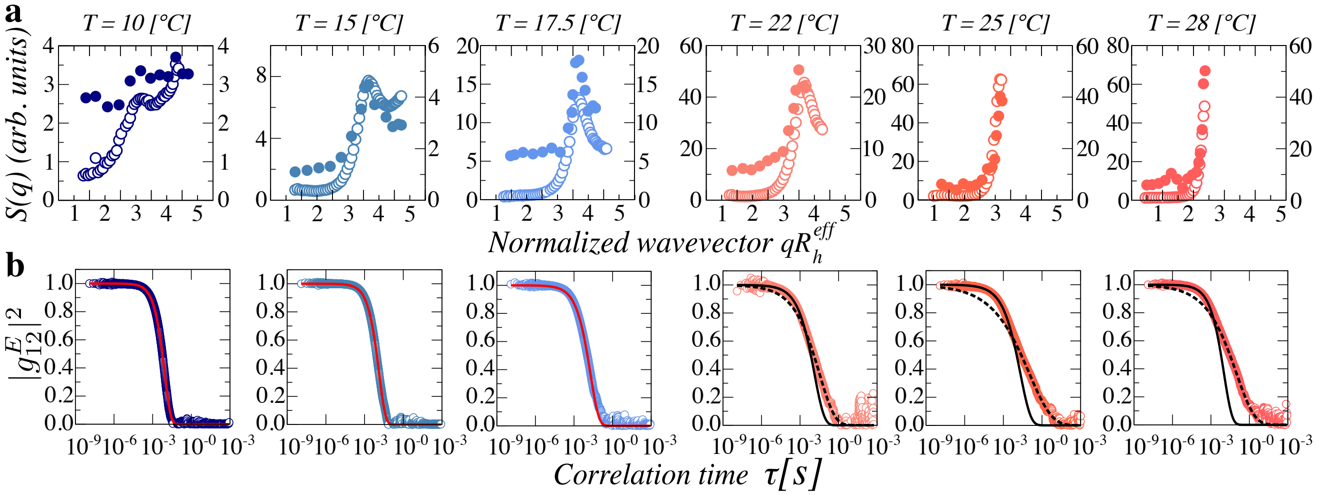

Figure S3: Experimental results for $\zeta = 3.0$. **a**, Measurable structure factor $S(q)$ (empty symbols), and normalized structural relaxation time $\tau_\alpha(q)/\tau_0$ (full symbols), with τ_0 the diffusive time scale obtained in dilute conditions. **b**, Square of the field correlation function $\left|g_{12}^E\right|^2$ as function of the correlation time τ, measured at q^*. Solid lines are fits to short-time stretched exponentials, while dashed lines represent fits to the long-time stretched exponentials. Corresponding panels in **a** and **b** are data at different temperatures. Note that while at low T, the correlation function is well fit by a single stretched exponential, for higher T, two stretched exponentials are required to describe the short-time and long-time dynamics.

SIV. MAPPING EXPERIMENTS TO SIMULATION MODEL

Table S1: Mapping between experiments and simulation model. Temperature, T, experimental ratio between the radius of gyration R_g and the hydrodynamic radius R_h, and associated simulation details. The overall particle size is σ_{eff}, core size is σ_{core} and intermediate shell size is σ_{mid}.

Experimental		Simulations		
$T[^\circ C]$	R_g/R_h	σ_{eff}	$\sigma_{core} = 2R_g$	$\sigma_{mid} = 0.5\,(\sigma_{eff} + \sigma_{core})$
10	0.572	1	0.574	0.787
15	0.619	1	0.621	0.811
17.5	0.669	1	0.670	0.835
22	0.673	1	0.676	0.838
28	0.664	1	0.676	0.838
32	0.686	1	0.684	0.842

SV. STATIC-DYNAMIC DECOUPLING IN SIMULATIONS FOR OTHER VALUES OF U_{mid}

We show here simulation results for $U_{mid} = 20k_BT$ and $U_{mid} = 200k_BT$; see Figs. Fig. S4 and Fig. S5. Panels (a) in these two figures show the corresponding pair potentials for four representative single-particle softnesses, which we associate to four experimental temperatures. Larger values of U_{mid}, for fixed values of $U_{corona} = 20\,k_BT$ and $U_{core} = 10^4\,k_BT$, correspond to stiffer particles. This, correspondingly, leads to a more pronounced static-dynamic coupling, as shown in Fig. S4(b) and Fig. S5(b). Concomitantly, $F_c(q^*,\tau)$ begins to exhibit a double decay at lower T; see Fig. S4(c) and Fig. S5(c).

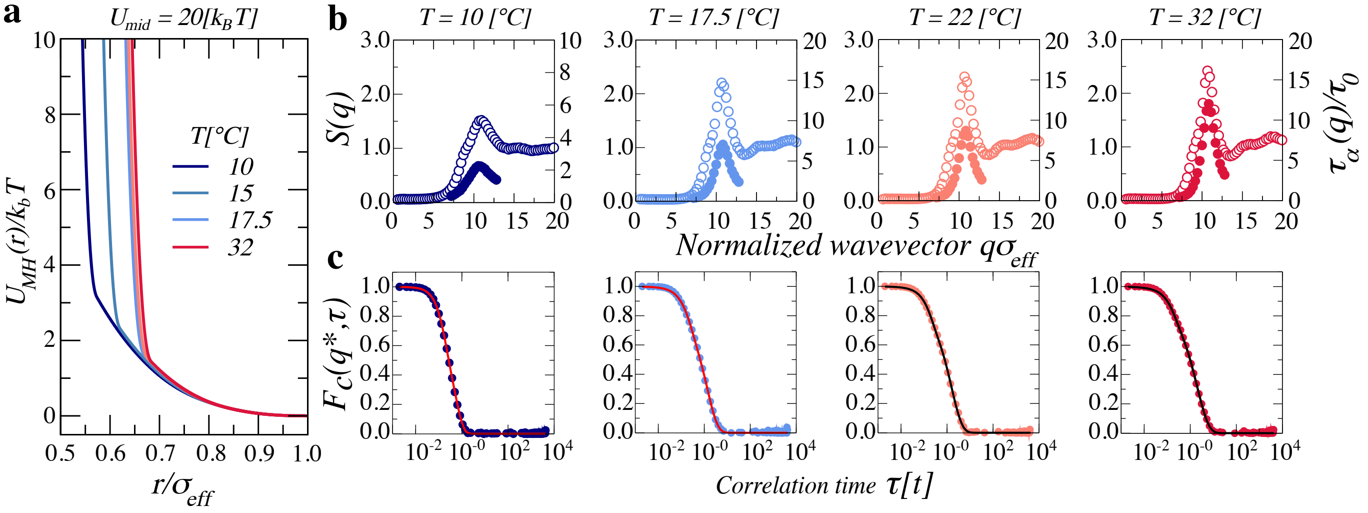

Figure S4: Numerical results for $U_{mid} = 20k_BT$ at $\zeta = 1.8$. **a**, Interaction potential as a function of T. **b**, Static structure factor $S(q)$ with empty symbols, and normalized structural relaxation time $\tau_\alpha(q)/\tau_0$ with full symbols; **c**, Dynamic structure factor $F_c(q^*,t)$, represented with empty symbols and computed at the wavevector q^* where $S(q)$ is maximum. Solid lines are the fits with one stretched exponential function at $T = 10°C$ and a sum of two stretched exponential functions at $T \geq 17.5°C$.

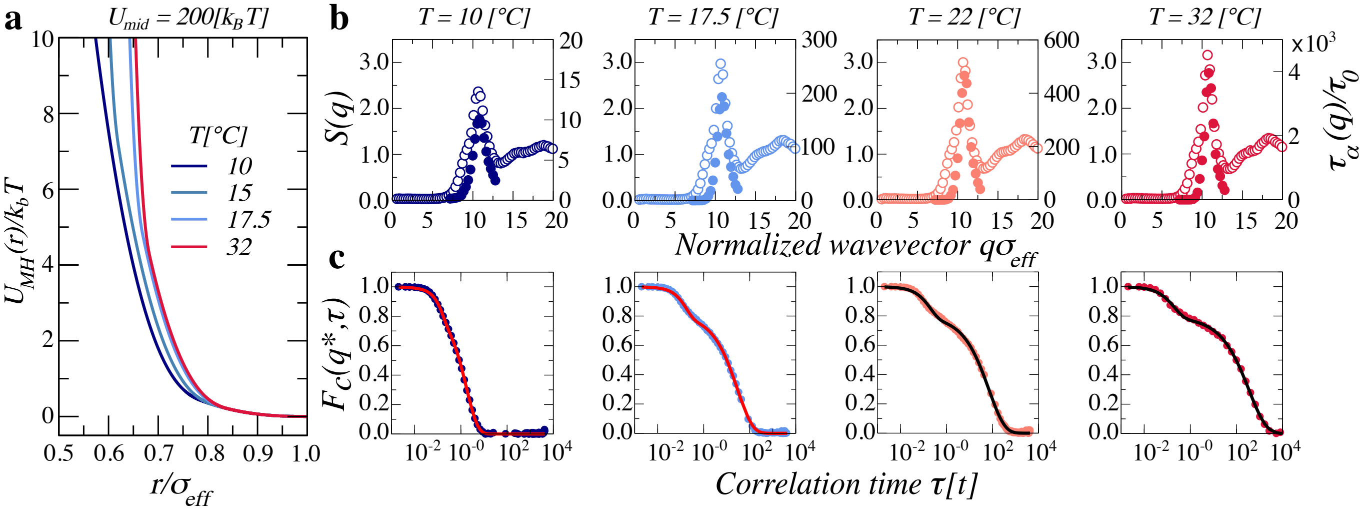

Figure S5: Numerical results for $U_{mid} = 200k_BT$ at $\zeta = 1.8$. **a**, Interactions potential as a function of T. **b**, Static structure factor $S(q)$ with empty symbols, and normalized structural relaxation time $\tau_\alpha(q)/\tau_0$ with full symbols; **c**, Dynamic structure factor $F_c(q^*,t)$, represented with empty symbols and computed at the wavevector q^* where $S(q)$ is maximum. Solid lines are fits to a sum of two stretched exponential functions at $T \geq 17.5°C$.

SVI. FITTING PARAMETER VALUES FROM THE MODIFIED ARRHENIUS PLOT

We show in Tables S2 and S3, the values of the fitting parameters associated to the simulation and experimental data shown in Fig. 4, respectively. For the system at $T = 10°C$, the ζ-dependence of the structural relaxation time is described by an Arrhenius exponential law, $\tau_\alpha(q^*)/\tau_0 \sim \exp[A\zeta]$, with A a constant. At higher T, the data is well described by a Vogel-Fulcher-Tamman law, $\tau_\alpha(q^*)/\tau_0 \sim \exp[A\zeta/(\zeta_0 - \zeta)]$, with A and ζ_0 constant parameters. Note how ζ_0 sets the ζ where τ_α diverges. We find that ζ_0 slightly decreases with increasing T, indicating an earlier approach to the glass for stiffer particles. The key, however, is the change in behavior with increasing T, suggesting a change from an apparently strong to an apparently fragile approach to the glass; apparent here refers to the fact that these conclusions are reached when plotting τ_α in terms of ζ, which at the high particle densities considered here, is different from the actual volume fraction ϕ.

Table S2: Values of the fitting parameters of the simulation $\tau_\alpha - \zeta$ curves for the case $U_{mid} = 100\,k_BT$.

Simulation results			
$T\,[°C]$	Theoretical model	A	ζ_0
10	Arrhenius model	0.909	-
15	VFT law	0.513	2.821
17.5	VFT law	0.614	2.302
22	VFT law	0.551	2.206
32	VFT law	0.437	2.094

Table S3: Values of the fitting parameters of the experimental $\tau_\alpha - \zeta$ curves.

Experimental results			
$T\,[°C]$	Theoretical model	A	ζ_0
10	Arrhenius model	1.156	-
22	VFT law	1.007	4.693

SVII. THE $\zeta - \phi$ CONNECTION AND THE APPROACH TO THE GLASS

To estimate ϕ in the simulations, we use a Voronoi tessellation [1], a technique that subdivides space into cells of volume V_v based on the positions of each particle in the system. A Voronoi cell is determined by the distance to its nearest neighbors. In cases where $V_v < V$, where V is the volume associated to the particle diameter σ_{eff}, there is overlap with other particles, and V_v represents the space occupied by the particle due to the deformation/shrinking required by the presence of nearby particles. In contrast, when $V_v > V$, the particle is not in contact with nearby particles and it is not considered deformed; the volume it occupies is then simply V.

We illustrate the situation for a system with $U_{mid} = 100\,k_BT$, at $\zeta = 2.0$ and $T = 10°C$. A snapshot of the system of particles with their associated volume is shown in Fig. S6(a); significant overlap can be observed. The Voronoi cells obtained from the particle positions are shown in Fig. S6(b). Here we define $\langle \overline{V_v} \rangle$ as the average Voronoi volume, with the upper bar indicating an average over particles and $\langle ... \rangle$ indicating an average over different snapshots. The associated volume fraction $\phi_v = \zeta \langle \overline{V_v} \rangle / \overline{V}$ increases with ζ, and saturates at a value of 1 for sufficiently high ζ, as shown in Fig. S6(c). Using ϕ_v as a control parameter, we then find that τ_α exhibits universal behavior irrespective of T, and thus of particle softness. This suggests the apparent fragility variation with softness is indeed apparent, as no variation is observed when plotted in terms of a somewhat realistic estimate of volume fraction; this is consistent with the theoretical model proposed in Ref. [2], and with the experimental and numerical results reported in Ref. [3, 4].

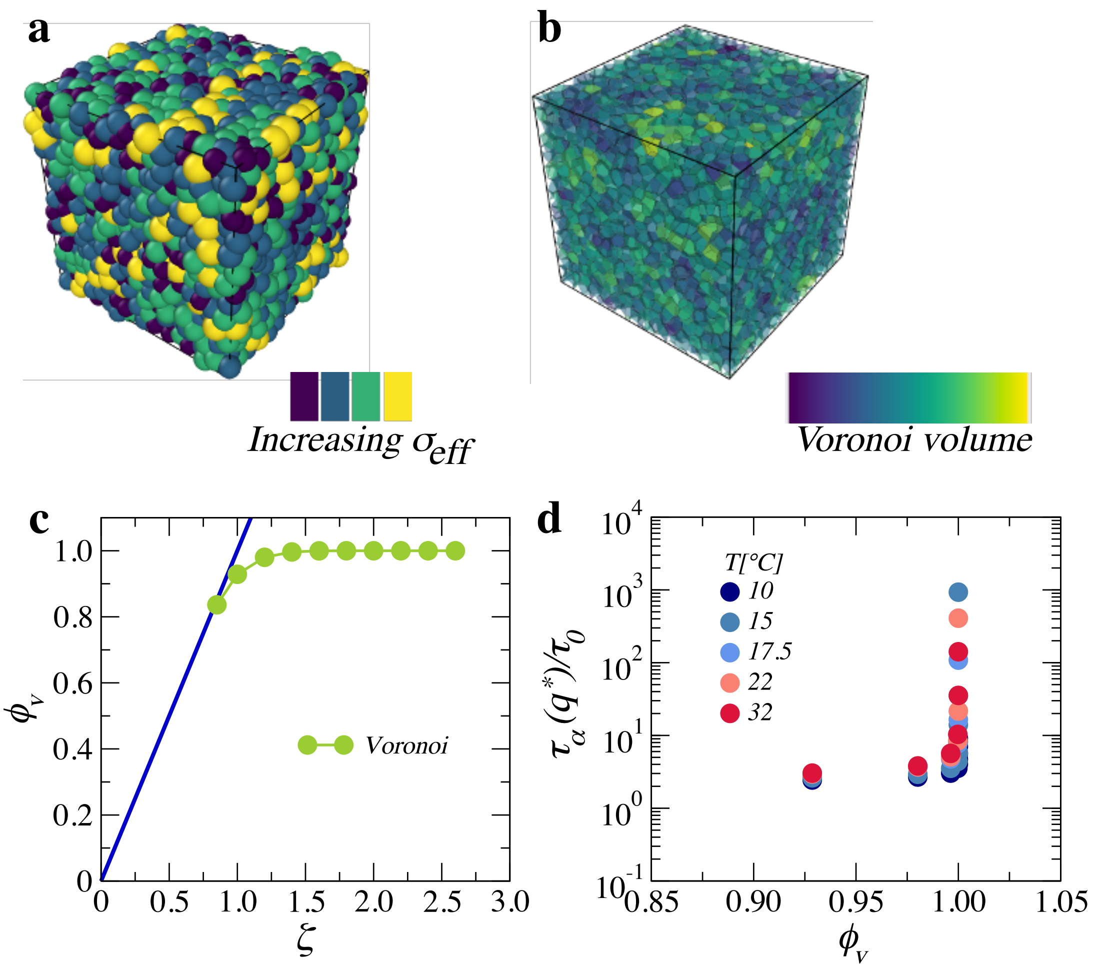

Figure S6: $\zeta - \phi$ relation using Voronoi tessellations. **a**,**b**, Snapshots of the particles in the simulation box. In **a**, the volume shown is V, while in **b** it is V_v. The system is at $\zeta = 2.0$ and $T = 10$°C. **c**, Mapping between the Voronoi volume fraction ϕ_v and the generalized volume fraction ζ. The line indicates a linear relation between both quantities. **d**, Normalized relaxation time as a function of ϕ_v, for $U_{mid} = 100 k_BT$.

[1] Rycroft, C. Voro++: A three-dimensional voronoi cell library in c++. Tech. Rep., Lawrence Berkeley National Lab.(LBNL), Berkeley, CA (United States) (2009).

[2] Van Der Scheer, P., Van De Laar, T., Van Der Gucht, J., Vlassopoulos, D. & Sprakel, J. Fragility and strength in nanoparticle glasses. *ACS nano* **11**, 6755–6763 (2017).

[3] Philippe, A.-M. *et al.* Glass transition of soft colloids. *Physical Review E* **97**, 040601 (2018).

[4] Higler, R. & Sprakel, J. Apparent strength versus universality in glasses of soft compressible colloids. *Scientific Reports* **8**, 16817 (2018).
